# Clinical Evidence for the Effects of Manual Therapy on Cancer Pain: A Systematic Review and Meta-Analysis

**DOI:** 10.1155/2021/6678184

**Published:** 2021-02-05

**Authors:** Chongjie Yao, Yanbin Cheng, Qingguang Zhu, Zhizhen Lv, Lingjun Kong, Min Fang

**Affiliations:** ^1^Yueyang Hospital of Integrated Traditional Chinese and Western Medicine, Shanghai University of Traditional Chinese Medicine, Shanghai, China; ^2^Research Institute of Tuina, Shanghai Academy of Traditional Chinese Medicine, Shanghai, China; ^3^College of Acupuncture and Tuina, Shanghai University of Traditional Chinese Medicine, Shanghai, China

## Abstract

**Objective:**

This meta-analysis aimed to evaluate the effects of manual therapy (MT) on cancer pain, so as to provide clinical evidence for application.

**Methods:**

Five English and Chinese databases were searched until February 29, 2020, for randomized controlled trials (RCTs) of MT for cancer pain. Articles published in the English or Chinese language were included. Two authors independently reviewed all articles and extracted the data, and any disagreements in the above process were discussed with other reviewers until the authors reached consensus. Review Manager 5.3 was used to calculate the effect size and 95% confidence intervals. This review was registered in PROSPERO, number CRD42020172053.

**Results:**

The intensity of cancer pain is our primary outcome measure, and compared with standard care, MT can significantly relieve the pain of patients with cancer (SMD, 0.63; 95% CI [0.18, 1.08]; *P*=0.006 < 0.01); the effects of MT plus active activity were significantly different from AT alone (SMD, 0.79; 95% CI [0.28, 1.30]; *P*=0.002 < 0.01); there was no statistical difference in the efficacy of MT and AT alone (SMD, -0.24; 95% CI [-1.09, 0.62]; *P*=0.53 > 0.05). In other related symptoms, the above evidence cannot support that MT had a good effect on fatigue (SMD, 0.77; 95% CI [-0.09, 1.63]; *P*=0.08 > 0.05), nausea (SMD, 0.24; 95% CI [-0.00, 0.48]; *P*=0.05), anxiety (SMD, 0.76; 95 % CI [-0.32, 1.84]; *P*=0.17 > 0.05), and depression (SMD, 0.67; 95 % CI [-0.28, 1.62]; *P*=0.17 > 0.05); however, MT intervention can improve physical function (*n* = 271; SMD, 0.35; 95 % CI [-0.04, 0.74]; *P*=0.04 < 0.05) and global well-being (SMD, 0.50; 95 % CI [0.02, 0.98]; *P*=0.04 < 0.05). In addition, MT had a significant effect on pain relief (SMD, 0.52; 95% CI [0.03, 1.01]; *P*=0.04 < 0.05) and improvement of physical function (SMD, 0.28; 95% CI [0.02, 0.53]; *P*=0.03 < 0.05) even after a period of time after treatment.

**Conclusion:**

MT was an effective intervention, which may have immediate effect on cancer pain and may improve physical function and global well-being. In the view of follow-up effects, MT had good effects for the reduction of pain and the recovery of physical function. However, because of limitations, the seemingly promising results should be interpreted with caution.

## 1. Introduction

With the rapid development of modern medicine, the cure rate of many diseases has increased considerably, but tumor is still the main killer affecting human health [1]. Constantly updated anticancer methods and pharmacologic agents significantly increased the survival rate of patients with malignant tumors, but their quality of life (QoL) was not obviously improved [2]. Continuous pain not only affects the physical health of cancer patients but also leads to severe anxiety, depression, insomnia, fatigue, and other symptoms [3]. Though the three-step analgesic ladder for managing cancer pain provided by the World Health Organization has been widely used in clinical practice [4], insufficient ability of pain assessment [5, 6], adverse reactions of analgesic drugs, and rising health costs make government organizations have to seek nonpharmacologic treatment [7]. In the 2019 version of adult cancer pain guidelines [8], the National Comprehensive Cancer Network (NCCN) has integrated a large number of nonpharmacologic therapies for cancer pain including massage, acupressure, acupuncture, psychological support, and exercise.

Manual therapy (MT), as complementary and alternative therapy, is skilled hand manipulations, including massage, chiropractic, osteopathic medicine, and others. MT was widely applied in many countries intended to improve soft tissue movement restriction, relieve pain, and promoting psychological well-being. Several studies reported that MT showed beneficial improvements in cancer-related pain and emotional problems [9, 10]. However, some reviews indicated that there was insufficient evidence on the effect of MT in relieving cancer pain [11]. The effects of MT for cancer pain and related symptoms are controversial.

In the past decade, many cancer sufferers used MT as a complementary therapy, to not only relieve pain but also promote psychological well-being [12]. And some high-quality randomized controlled trials (RCTs) were published [13–15], which paid more attention to the follow-up effects of MT for cancer patients. In China, MT, named Tuina, was widely applied to relieving pain in patients [16, 17], but the related studies did not get sufficient attentions in the previous reviews. In this study, more rigorous RCTs published in recent years and Chinese studies were included.

The current systematic review was aimed to examine the evidence on the effect of MT for cancer pain and related psychological well-being. The following questions are focused: (1) the effectiveness of MT in relieving cancer pain compared with standard care or other nonpharmacologic treatments; (2) the effects of MT in promoting psychological well-being by improving depression, anxiety, nausea, and others; (3) the follow-up effects of MT after the final treatment.

## 2. Methods

This study followed the guidelines of Preferred Reporting Items for Systematic Reviews and Meta-Analyses (PRISMA). This review was registered in PROSPERO (registration number: CRD42020172053).

### 2.1. Search Strategy

Five databases and reference lists were searched for RCTs published until February 29, 2020. English databases included PubMed and EMBASE, and Chinese databases included China National Knowledge Infrastructure, VIP Database for Chinese Technical Periodicals, and Wanfang Data. The search strategy consisted of four components: disease diagnosis (“neoplasia” OR “tumor” OR “cancer” OR “malignancy” OR “carcinoma”), clinical condition (“pain” OR “analgesia” OR “symptom relief”), intervention method (“massage” OR “Tuina” OR “zone therapy” OR “reflexology” OR “Rolfing” OR “bodywork” OR “manipulation” OR “chiropractic” OR “osteopathic” OR “physical therapy” OR “motion therapy”), and study type (randomized clinical trial). Appropriate keywords from MeSH headings were used in combination to develop searches by titles or abstracts to establish the eligibility of the studies. In addition, reference lists from all relevant articles were reviewed to make sure no RCTs were missed.

### 2.2. Study Selection

Two authors (C. Y. and L. K.) independently reviewed all articles by titles and abstracts, or full text if necessary, to evaluate their eligibility for inclusion. Articles published in the English or Chinese language were included if they were RCTs (excluding crossover design) investigating the association of MT with cancer pain. Patients with various types (breast cancer, lung cancer, colon cancer, etc.) of cancers were included without any restrictions on the age, gender, race, clinical status, and duration of cancer, but the baseline data must show no significant statistical difference between the experiment group (EG) and control group (CG) in each independent RCT. Eligible EG interventions were any technique of MT, including massage, osteotomy, chiropractic, acupressure, reflexology, trigger point therapy, and other physical therapies operated only by hands, compared to placebo, standard care, and any active treatments not related to MT as the CG. In addition, cancer pain in this article included pain directly caused by the development of cancer, chronic pain associated with cancer treatment, and acute pain after surgery.

The primary outcome of interest was pain, which can be measured by the Numerical Rating Scale (NRS), the Visual Analog Scale (VAS), the Brief Pain Inventory (BPI), and any other validated instrument. The secondary outcomes were QoL, functional improvement, negative emotions, and other cancer-related symptoms, for which no restriction set on the type of tool used in the studies as there were no universally accepted tools available. These symptoms had clear diagnostic criteria in related RCTs and were assessed by different scales, such as Short Form-36 questionnaire (SF-36), Functional Assessment of Cancer Therapy-Breast (FACT-B), Brief Fatigue Inventory (BFI), and others.

Studies were excluded if any of the following were identified: (1) the study only reported improvement rates and no other specific data to refer to; (2) the use of MT was not the single variable between intervention of the EG and CG, because other factors in the experiment may affect the results (e.g., music and acupuncture); (3) the intervention of CG contained MT, because the effects of MT could not be assessed; (4) the language of articles was neither English nor Chinese.

### 2.3. Data Abstraction and Methodological Quality Assessment

All the data were independently extracted by two reviewers (C. Y. and L. K.) in the mentioned databases according to predefined criteria, including first author, country of the study, year of the study, clinical situation, sample size, mean age of participants, duration of treatments, follow-up time, interventions of the EG and CG, outcome measures, and results. However, some less frequent outcome measures were not analyzed to better integrate the data (*n* < 3). Studies were excluded if they did not provide complete data needed to calculate the effect size. If there were multiple assessment time points, the time point of the last postintervention was chosen. To ensure rigor in the data abstraction process, the two reviewers also independently checked all the records to minimize bias.

All RCTs included in the study were assessed independently by two reviewers according to the physiotherapy evidence database (PEDro) scale, which is reported to have excellent reliability for RCTs of the physiotherapy [18]. The risk of bias of each study was assessed through the generation of a score, which was calculated by 11 items in the PEDro scale. Each item is scored as either 1 or 0 according to whether the item is met or not. However, the first item is not used to calculate the final score, so the total score ranges from 0 to 10. A higher score indicates better methodological quality, but it has been reported a score of at least 6 is considered a high-quality study [19]. We contacted the study authors if more information is needed, and any disagreements in the above process were discussed with another reviewer (Y. C.) until the authors reached consensus.

### 2.4. Data Synthesis and Statistical Analysis

The meta-analysis was performed by calculating the effect size and 95% confidence intervals (CI) in the Review Manager 5.3. To assess the effects of MT on each outcome measure in the meta-analyses, we used the mean changes in outcomes between the end of final intervention and the baseline, which showed the difference between the EG and CG. The standardized mean difference (SMD) was used because different scales were applied to evaluate the outcomes, including NRS, VAS, and BPI. For studies with more than one CG, the results were split into comparisons between the EG and each CG. Statistical heterogeneity was assessed using *I*^2^, and the value of which more than 50% was determined as a high level of heterogeneity. If *I*^2^ <  50% in the results, we used a fixed effect model, otherwise a random effects model was used. A funnel plot was used to analyze bias.

## 3. Results

**Figure 1:**
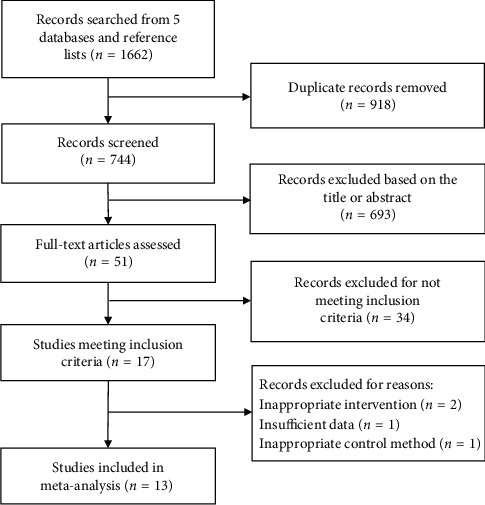
Study selection process.

1662 records were searched from 5 databases, and reference lists were included. After removing duplicates and screening eligibility by title and abstract, fifty-one articles were included to be fully assessed. Thirty-four studies were excluded because they did not meet the inclusion criteria, and we selected 17 eligible articles. In the process of exclusion, the studies were excluded due to inappropriate intervention (*n* = 2) [20, 21], insufficient data (*n* = 1) [22], and inappropriate control method (*n* = 1) [23]. In the end, a total of 13 studies were included in our meta-analysis, including 11 English articles and 2 Chinese. The study selection process is summarized in Figure 1.

### 3.1. Study Characteristics

A total of 13 eligible studies, ranging from 2000 to 2019, evaluated the effects of MT on cancer pain. Eleven hundred participants, including 556 in the EG and 544 in the CG, with the mean age of 55.23, were conducted, respectively, in the USA, Germany, Italy, the UK, China, and other countries. Six studies focused on a specific kind of cancer (5 breast cancer [13–15, 24, 25] and 1 gastric or liver cancer [26]), and the remaining 7 RCTs involved any type of cancer in any stage [27–33].

Two studies [26, 33] observed the short-term effects of MT on cancer pain, and the treatment duration was 2 days and 3 days, respectively, so no follow-up was conducted. The other 11 studies [13–15, 24, 25, 27–32] lasted from 2 weeks to 3 months, 5 [13–15, 25, 29] of which involved follow-up for 6 weeks to 3 months. One study [29] claimed that the results of follow-up would be reported separately, but we did not find them. Of the 13 RCTs, one study [33] only observed cancer pain and other studies involved cancer-related side effects such as anxiety, depression, and fatigue.

MT in the studies mainly included massage therapy [15, 25, 27–29, 32], myofascial therapy [14, 24, 30], foot reflexology [13, 26], osteopathic manipulative treatment [31], and acupressure [33]. The control therapies contained standard care [13, 15, 26, 27, 30, 32, 33] and active therapies (AT) including physical therapy [14, 31], kinesiotherapy [24, 25], reading therapy [29], and psychological support [28]. The frequency of intervention was from twice a day to once a week, and each intervention method lasted from 10 to 50 minutes. When assessing the effects of MT, seven studies [13, 15, 26, 27, 30, 32, 33] compared the efficacy differences between MT and standard care. In addition, four studies [14, 24, 25, 31] compared MT plus AT and AT alone, and two studies [28, 29] compared MT with AT. The details of all studies are summarized in Tables 1 and 2.

### 3.2. Methodological Quality

The methodological quality of the studies was accessed in Table 3. According to the PEDro scale, all the studies received a score of 6 or more, indicating they were considered to be of high quality. However, five studies [15, 27, 31–33] were at the limit of the cutoff with scores of 6, and the reason for which was that although random assignment of patients was adopted, they did not use the appropriate method of assignment concealment. The most common defect was the lack of blinded therapists and blinded subjects, but this situation cannot be considered as a defect because it was difficult to implement in the study, and all the studies used blinded assessors. The highest score among the included studies was 9, which was for the only study that blinded the participants [14]. Most studies did not use intention-to-treat analysis because they cancelled the dropout data in the last results. In other items on a PEDro scale, the studies showed high methodological quality, including similarity between groups at baseline, less than 15% dropouts, between-group statistical comparisons, and point measures and variability data.

### 3.3. Quantitative Data Synthesis

Pain is the primary outcome to be analyzed, and we further analyzed the subgroups according to the different intervention methods of the CG. In addition, we also studied the effects of MT on other cancer-related side effects mentioned in RCTs included.

#### 3.3.1. The Effects of MT on Cancer Pain

**Figure 2:**
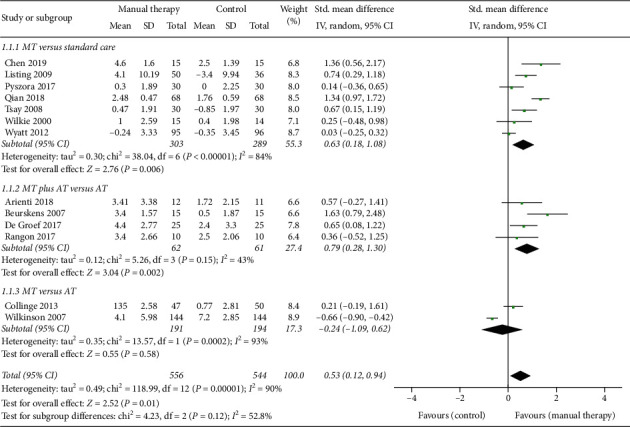
Forest plot of the effects of manual therapy on cancer pain.

*(1) MT versus Standard Care*. As shown in Figure 2, 7 (54%) studies [13, 15, 26, 27, 30, 32, 33] took standard care as the CG to observe the effects of MT on cancer pain. In these RCTs, only one study [27] showed that there was no significant difference between the EG and CG. Our analysis demonstrated that, compared with standard care, MT can significantly relieve the pain of patients with cancer (*n* = 592, SMD, 0.63; 95% CI [0.18, 1.08]; *P*=0.006 < 0.01). From the existing evidence, acupressure may have a good effect on cancer pain. Chen et al. [33] and Qian et al. [32] significantly reduced pain after pressing on acupoints related to symptoms.

*(2) MT plus AT versus AT*. In Figure 2, 4 (31%) RCTs [14, 24, 25, 31] observed whether the addition of MT would increase the efficacy of AT alone. One study [31] suggested that MT combined with passive mobility, active exercises, and walk could not significantly increase the efficacy. However, through integrating the results of 4 studies, we believed that the effects of MT plus AT can be significantly different from AT alone (*n* = 123, SMD, 0.79; 95% CI [0.28, 1.30]; *P*=0.002 < 0.01).

*(3) MT versus AT*. In Figure 2, the last 2 (15%) studies compared the effects of MT alone and AT on cancer pain. In one study, 288 patients were observed, and it was found that the effects of MT were worse than psychological support, although there was no statistical difference. Another study with a smaller sample size [29] suggested that MT was more effective in relieving pain than reading therapy. Through comprehensive analysis, we believed that the existing evidence cannot demonstrate the efficacy of MT is better than AT alone (*n* = 385, SMD, −0.24; 95% CI [−1.09, 0.62]; *P*=0.53 > 0.05).

#### 3.3.2. The Effects of MT on Other Related Symptoms

**Figure 3:**
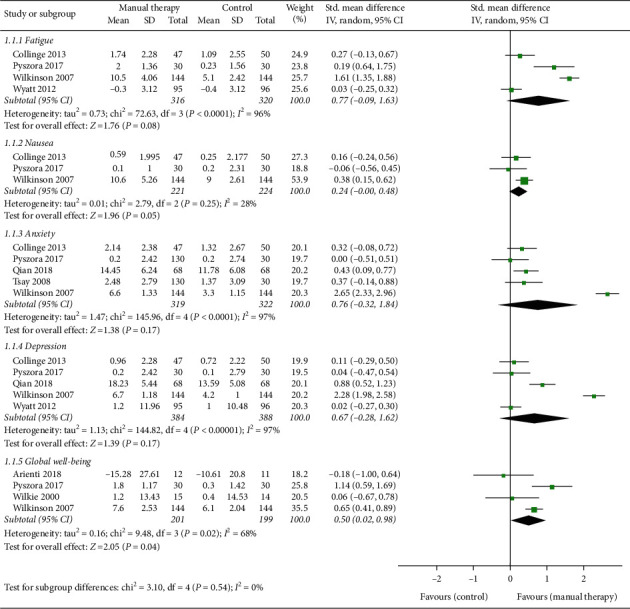
Forest plot of the effects of manual therapy on other related symptoms.

*(1) Fatigue*. As shown in Figure 3, fatigue, another major physical symptom related to cancer besides pain, was proved in 3 studies [13, 28, 29] that there was no significant difference between the EG and CG on relieving symptom. Pyszora et al. [30] suggested that compared with standard care, MT can significantly improve the fatigue symptom of patients, which was different from other RCTs. In addition, one study [15] which was not included in the analysis due to the lack of definite data indicated that the results were also in favor of EG but did not reach statistical significance. Therefore, we cannot support statistically that MT intervention can have a better effect (*n* = 636, SMD, 0.77; 95% CI [−0.09, 1.63]; *P*=0.08 > 0.05), although MT may have a positive result from the clinical evidence.

*(2) Nausea*. In Figure 3, 3 studies [28–30] observed the improvement of MT on nausea. Only one study [29] reported that MT could improve symptoms statistically better than reading therapy. In another study [30], the intervention of MT was less effective than standard care, although the difference was not statistically significant. Therefore, based on the existing evidence, we can only consider that MT intervention may have a weak advantage in nausea (*n* = 445, SMD, 0.24; 95% CI [−0.00, 0.48]; *P*=0.05).

*(3) Anxiety*. In Figure 3, one study [32] suggested that MT can significantly improve the anxiety of cancer patients. However, in most (80%) studies [26, 28–30], although MT may relieve anxiety, the difference was not statistically significant. Therefore, the above evidence cannot support that MT had a good effect on anxiety (*n* = 641, SMD, 0.76; 95% CI [−0.32, 1.84]; *P*=0.17 > 0.05).

*(4) Depression*. In Figure 3, it was demonstrated that MT had no effect on depression (*n* = 772, SMD, 0.67; 95% CI [−0.28, 1.62]; *P*=0.17 > 0.05), although all the studies reported that MT intervention may have a positive effect on symptoms. Only Qian et al. [32] demonstrated that acupressure could significantly relieve depression of patients compared with standard care.

*(5) Global Well-Being*. In Figure 3, 3 studies [27, 28, 30] suggested that MT could improve global well-being compared with the CG, although 2 of them [27, 28] did not have statistical difference. However, in the study of Pyszora et al. [30], the researchers achieved significant effects through MT intervention. In addition, another study [31] added MT on the basis of exercise therapy, making the curative effect worse, although the difference was not statistically significant. Therefore, our evidence supported that MT intervention can increase the global well-being of cancer patients (*n* = 400, SMD, 0.50; 95% CI [0.02, 0.98]; *P*=0.04 < 0.05).

#### 3.3.3. The Effects of MT on Physical Function

**Figure 4:**
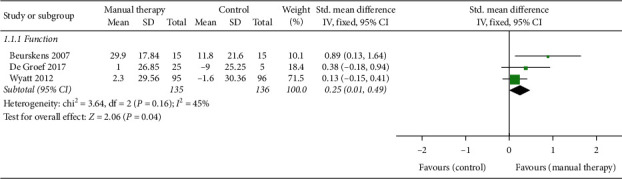
Forest plot of the effects of manual therapy on physical function.

As shown in Figure 4, the effects of MT on the recovery of physical function were observed in 3 studies [13, 14, 25]. Although the results of De Groef et al. [14] supported the positive effects of MT, there was no statistical difference. Wyatt et al. [13] studied the effects of foot reflexology on physical function, demonstrated that MT can improve the function, while standard care can reduce the original function, and the difference was statistically significant. In another study, Beurskens et al. [25] added massage to home exercise, which significantly improved the efficacy. Therefore, our evidence supported that MT got better effect in improving physical function (*n* = 271, SMD, 0.35; 95% CI [‒0.04, 0.74]; *P*=0.04 < 0.05).

#### 3.3.4. The Follow-Up Effects of MT

**Figure 5:**
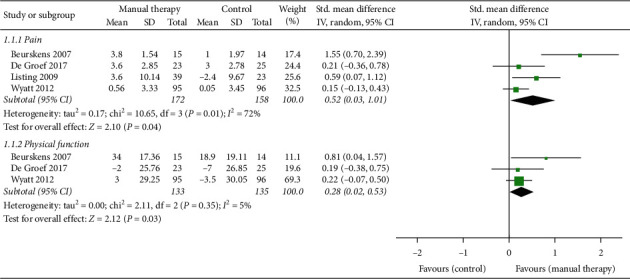
Forest plot of the follow-up effects of manual therapy.

As shown in Figure 5, our study was the first time to analyze the follow-up effects of MT. During the follow-up, the main observation was pain and physical function, and the time was 6 weeks to 3 months. The changes of pain after the end of intervention were observed in four studies [13–15, 25]. The results suggested that the effects of MT on cancer pain were still beneficial in the follow-up, although the results of 2 studies [13, 14] were not statistically significant. In the study of Listing et al. [15], standard care alone exacerbated pain during follow-up, while MT significantly improved the situation. Therefore, current evidence demonstrated that MT had a significant effect on pain relief even after a period of time after treatment (*n* = 330, SMD, 0.52; 95% CI [0.03, 1.01]; *P*=0.04 < 0.05).

The follow-up effects of MT on physical function were studied in 3 studies [13, 14, 25]. MT had a positive effect on functional recovery, but the result of one study [14] was not statistically significant. In the other 2 studies [13, 25], MT had significantly improved the function in the evaluation after the intervention, and the improvement was maintained until the follow-up. Therefore, based on the above research studies, we can consider that MT was conducive to the future recovery of physical function, and the results were statistically significant (*n* = 268, SMD, 0.28; 95% CI [0.02, 0.53]; *P*=0.03 < 0.05).

#### 3.3.5. Risk of Bias

**Figure 6:**
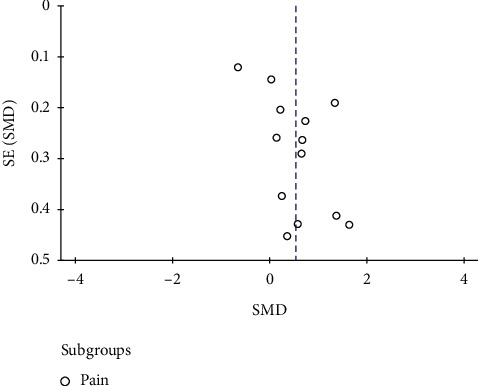
Funnel plot of the risk of bias.

In Figure 6, no obvious asymmetric distribution of the trials was observed in a funnel plot, but the possibility of publication bias cannot be ruled out. The small sample size may be a major reason for this possible bias.

## 4. Discussion

The meta-analysis included 13 RCTs with 1100 patients, to provide an updated synthesis of the current evidence for the effects of MT on cancer pain and fill some research gaps remained to be addressed in the past. We not only brought into more studies and pay attention to follow-up after intervention, but also searched Chinese RCTs, which were not analyzed in the past.

### 4.1. Analysis of Research Results

Consistent with results of a few systematic reviews and meta-analyses in the past [34, 35], evidence was found of an association between MT and reduction in pain, especially when compared with standard care or MT is added on the basis of AT. However, more previous studies supported that MT had no beneficial effects on cancer pain relief. In the Cochrane Database of Systematic Reviews, Shin et al. [36] held the view that there was a lack of evidence for effectiveness of massage on symptom relief of cancer, and most studies were unreliable and did not report key outcomes. In addition, Boyd et al. [12] and Pan et al. [37] suggested that the evidence was not enough to demonstrate that massage can reduce cancer pain compared to no treatment or sham control. There were also a few studies suggested the seemingly promising results should be interpreted with caution because of limitations [11].

Based on the discussion of pain, we further analyzed the other cancer-related side effects involved in RCTs included, which are often accompanied by pain. In other symptoms, the effects of MT were mainly reflected in improving global well-being, which was never been mentioned before. Consistent with the results of previous studies [36, 37], MT showed no significant difference compared with the CG in anxiety and depression, but we found that MT had a weak advantage in relieving nausea. In addition, fatigue, which was supported to be improved by MT in previous studies [36], did not reflex beneficial effect in our study, but it was reported positive results in most RCTs [15, 28–30].

We believed that the above differences were mainly due to the following reasons. First of all, this study analyzed massage, osteology, chiropractic, acupressure, reflexology, trigger point therapy, and any other physical therapy operated only by hands as MT, instead of studying them separately as in the past [9, 11], because it was impossible to completely separate them in practical application. Secondly, we included a part of Chinese RCTs [32, 33], although few Chinese studies in this field were of high quality. In addition, we did not include crossover design experiments because it may affect the final results, especially when we need to consider follow-up results. Finally, studies that did not involve pain were not included in the analysis of MT on other related symptoms, because pain relief was the main purpose of this study.

The current review, to our knowledge, was the first to evaluate the follow-up effects of MT for cancer pain. According to our results, it can be demonstrated that MT could significantly reduce cancer pain and improve physical function at the end of the intervention, and the significant effects can even last until the follow-up. Therefore, the intervention of MT not only had immediate and sustained analgesic effect but also brought great benefits for the future physical function recovery.

In addition, QoL is a frequent outcome in the RCTs, which we mentioned in Table 3. However, the results of QoL cannot be analyzed comprehensively because there was no unified standard for it. De Groef et al. [14] took QoL as a comprehensive index to evaluate physical function and mental function, and we made another analysis on the former. In the study of Listing et al. [15], QoL was divided into breast symptoms and arm symptoms, and the former was significantly improved after MT intervention. Rangon et al. [24] and Wyatt et al. [13] used FACT-B scale to evaluate QoL, so their research perspective was similar. European Organization of Research and Treatment of Cancer QoL Questionnaire (EORTC QLQ) was also applied to access QoL, but researchers chose different subprojects according to different research purposes [28, 31]. Beurskens et al. [25] used Sickness Impact Profile (SIP) to evaluate the physical disability of patients to demonstrate the significant improvement of QoL. In addition, Collinge and his colleagues [29] used the simple NRS with pain, fatigue, depression, and nausea as the subprojects of QoL, which were analyzed in our study. Therefore, the direct analysis of QoL in the past research was worth further discussion [38].

### 4.2. Limitations of the Review

There were several limitations in our study. First of all, substantial heterogeneity was observed which mainly owed to the application of different kinds of measurement methods. As our primary outcome measure, pain was evaluated by VAS or NRS in 9 (69%) studies [14, 24–27, 29, 31–33]. The two scales were similar because they measured pain on a score of 1 to 10, and the higher the score, the more severe the pain. Although the VAS score in some studies [14, 26] was 1–100, it was also converted to the maximum of 10 in our review, which did not affect the final result. However, some special scales were used in other research studies. In the study of Listing et al. [15], Short Form-8 Health (SF-8) survey was applied to evaluate pain, which contained one item for each of the eight concepts of the SF-36, and the pain of patients decreased with increasing scores. Other scales were not described in details here, but obviously different measurement methods were the main reason for high heterogeneity. Cancer, on the other hand, is so complex that various measures may describe different dimensions of symptoms, and pain types and treatments can also affect outcomes. In addition, although we thought it was difficult to distinguish different MT methods completely, the difference of MT technology, frequency, duration, and treatment courses may affect the heterogeneity. Therefore, more studies were needed to fully assess how these factors play a role in heterogeneity.

Secondly, the RCTs we included may have possible selection bias. The results did not change when we restricted the analysis to the methodological quality through the PEDro scale. Almost in all studies, both performance and response biases were possible since the lack of blinded therapists and blinded subjects, which were hard to avoid in practical treatment. However, the small sample size and low methodological quality of some of the included studies was worthy of our attention. In addition, although our funnel plot did not show obvious asymmetric distribution, it is difficult to interpret the results of publication bias due to such a small subset of studies. Thus, larger sample sizes and carefully planned designs are required for future analysis, as well as better monitoring of selected parameters.

Finally, the analysis of cancer-related side effects in this study was based on patients suffering from pain. Because pain was our primary outcome measure, a large number of RCTs that did not involve pain were not included in our study, which meant that many studies on fatigue, depression, anxiety, and other symptoms were ignored. On one hand, we mainly focused on cancer pain, so other studies that had nothing to do with pain were excluded; on the other hand, almost all studies of cancer pain involved other accompanying symptoms, which we cannot ignore. Moreover, there were few studies involving each symptom, because of many kinds of other cancer-related symptoms in different RCTs, which may have an impact on the results. In addition, only a few RCTs including follow-up studies were found, and the follow-up results in which were often similar to those after the intervention. Therefore, the current evidence only demonstrated that MT could improve cancer-related side effects on the basis of reducing cancer pain, but whether MT had long-term effects needed further study.

## 5. Conclusion

The current evidence demonstrated that MT was an effective intervention, which may have immediate effect on cancer pain and may improve physical function and global well-being. Although MT achieved positive results on fatigue, nausea, anxiety, and depression, the current evidence cannot support the effectiveness. In the view of follow-up effects, MT had good effects for the reduction of pain and the recovery of physical function. However, because of limitations, the seemingly promising results should be interpreted with caution.

It was necessary to establish relevant standards for the intervention of MT on cancer pain, such as frequency, duration, and course of treatment, to ensure the normalization of treatment. In addition, for some important outcome indicators, it was better to use a unified measurement method and added special scales if necessary.

## Figures and Tables

**Table 1:** Characteristics of studies of manual therapy on cancer pain.

First author, year, country	Clinical situation	Sample size	Mean age (year)	Duration	Followup	Outcome measures		
De Groef, 2017, Belgium [14]	Breast cancer (postoperation)	EG: 25	EG: 55.3			Primary	Pain (VAS↓)	
				3 months	3 months			Physical function
						Secondary	QoL (SF-36↑)	
		CG: 25	CG: 53.1					Mental function

Listing, 2009, Germany [15]	Breast cancer (I-II, postoperation)	EG: 50	EG: 57.6			Primary	Pain (SF-8↑)	
				5 weeks	6 weeks			Breast symptoms
						Secondary	QoL (EORTC QLQ-BR23↓)	
		CG: 36	CG: 61.4					Arm symptoms

Rangon, 2017, Brazil [24]	Breast cancer (postoperation)	EG: 10	EG: 55.4			Primary		
				5 weeks	No follow-up		Pain (NRS↓)	
		CG: 10	CG: 54.4			Secondary	QoL (FACT-B↑)	
							QoL (FACT-B↑)	

Wyatt, 2012, USA [13]	Breast cancer (III-IV, or I-II with metastasis or recurrence)	EG: 95	EG: 55.3			Primary		
							Physical function (SF-36↑)	
				5 weeks	6 weeks		Pain (BPI↓)	
		CG: 96	CG: 57.3			Secondary	Fatigue (BFI↓)	
							Depression (CES-D↓)	

Tsay, 2008, Taiwan [26]	Gastric or liver cancer (postoperation within 24 hours)	EG: 30				Primary	Pain (VAS↓)	
			59.8 ± 14.7	3 days	No follow-up			
		CG: 30				Secondary	Anxiety (HADS↓)	

Beurskens, 2007, Netherlands [25]	Breast cancer (postoperation)	EG: 15	EG: 53.7			Primary	Pain (VAS↓)	
				3 months	3 months		Physical function (DASH↓)	
		CG: 15	CG: 55.4			Secondary		
							QoL (SIP↓)	

Wilkie, 2000, USA [27]	Any type of cancers (advanced)	EG: 15				Primary	Pain (VAS↓)	
			64	2 weeks	No follow-up			
		CG: 14				Secondary	QoL (Graham's QoL↑)	Global well-being
						Primary	Anxiety (SAI↓)	

Wilkinson, 2007, UK [28]	Any type of cancers (any stage)	EG: 144	EG: 51.5				Depression (CES-D↓)	
								Pain
				10 weeks	No follow-up			
								Fatigue
						Secondary	QoL (EORTC QLQ-C30↓)	
		CG: 144	CG: 52.8					Nausea
								Global well-being

Collinge, 2013, USA [29]	Any type of cancers (any stage)					Primary	Anxiety (NRS↓)	
		EG: 47	54.7	4 weeks	16 weeks			
						Secondary	QoL (NRS↓)	Pain
		CG: 50						Fatigue
								Depression
								Nausea

Pyszora, 2017, Poland [30]	Any type of cancers (advanced)					Primary	Fatigue (BFI↓)	Pain
		EG: 30	EG: 72.4	2 weeks	No follow-up			Nausea
						Secondary	Symptoms (ESAS↓)	Depression
		CG: 30	CG: 69.3					Anxiety
								Global well-being

Arienti, 2018, Italy [31]	Any type of cancers (postoperation)					Primary	Pain (NRS↓)	
		EG: 12	EG: 76.5					
				4 weeks	No follow-up			Global well-being
						Secondary	QoL (EORTC QLQ-C30↓)	Financial difficulties
		CG: 11	CG: 76.5					Summary score

Qian, 2018, China [32]	Any type of cancers (advanced)	EG: 68	EG: 43.7	2 weeks	No follow-up	Primary	Pain (NRS↓)	
							Anxiety (SAS↓)	
						Secondary		
		CG: 68	CG: 45.1					
							Depression (SDS↓)	

Chen, 2019, China [33]	Any type of cancers (advanced)	EG: 15	EG: 55.3				Pain (NRS↓)	
				2 days	No follow-up	Primary		
		CG: 15	CG: 54.2					

EG: experiment group; CG: control group; VAS: Visual Analog Scale; QoL: quality of life; SF-36 : Short Form-36 Questionnaire; SF-8 : Short Form-8 Health Survey; EORTC QLQ: European Organization of Research and Treatment of Cancer QoL Questionnaire; NRS: Numerical Rating Scale; FACT-B: Functional Assessment of Cancer Therapy-Breast; BPI: Brief Pain Inventory; BFI: Brief Fatigue Inventory; CES-D: Center of Epidemiologic Studies-Depression Scale; HADS: Hospital Anxiety and Depression Scale; DASH: Disability of the Arm, Shoulder, and Hand Questionnaire; SIP: Sickness Impact Profile; SAI: State Anxiety Inventory; ESAS: Edmonton Symptom Assessment Scale; SAS: Self-Rating Anxiety Scale; SDS: Self-Rating Depression Scale. “↑” indicates that the higher the score was, the better the symptoms, and “↓” indicates that the lower the score was, the better the symptoms.

**Table 2:** Intervention process of studies of manual therapy on cancer pain.

First author, year, country	Intervention EG	Procedure EG	Intervention CG	Procedure CG
De Groef, 2017, Belgium [14]	18 sessions of a standard physical therapy program of 30 min (week 1–8 twice a week, week 9–12 once a week). 12 sessions of myofascial therapy of 30 min (once a week)	Physical therapy: shoulder mobilization; pectoral muscle stretching; exercise therapy. Myofascial therapy: myofascial release on active myofascial trigger points at the upper body, on myofascial adhesions in the pectoral, axilla, cervical region, diaphragm, and scars	18 sessions of a standard physical therapy program of 30 min (week 1–8 twice a week, week 9–12 once a week). 12 sessions of placebo treatment of 30 min (once a week)	Physical therapy: shoulder mobilization; pectoral muscle stretching; exercise therapy. Placebo: placements of hands up and down the upper body and arm on the affected side and lasted for 10–15 sec at one location

Listing, 2009, Germany [15]	10 sessions of classical massage of 30 min (twice a week)	Classical massage: massage of the back, neck, and head, consisted of Swedish techniques such as stroking, kneading, frictions, pressing on the trigger points, stretching the neck and the lumbar spine area, and depressing the shoulders and the hip area	Medical routine	No intervention, standard care

Rangon, 2017, Brazil [24]	10 sessions of kinesiotherapy of 50 min (twice a week). 10 sessions of ischemic compression of 90 sec (twice a week)	Kinesiotherapy: walk; neck active stretching, anterior and posterior chain of higher trunk; active mobilization of the cervical spine, upper limbs; relaxation exercises. Ischemic compression: pressing bilaterally on the myofascial trigger point centrally located in the upper trapezius muscle	10 sessions of kinesiotherapy of 50 min (twice a week)	Kinesiotherapy: walk; neck active stretching, anterior and posterior chain of higher trunk; active mobilization of the cervical spine, upper limbs; relaxation exercises

Wyatt, 2012, USA [13]	20 sessions of foot reflexology of 30 min (4 times a week)	Foot reflexology: stimulation of the nine essential breast cancer-specific reflexes with reflexology-specific deep thumb-walking pressure	Medical routine	No intervention, standard care

Tsay, 2008, Taiwan [26]	3 sessions of foot reflexotherapy of 20 min (once a day)	Foot reflexology: massage of digestive reflex zones of upper and lower abdomen, liver, spleen, gall bladder, duodenal, intestine, and colon	Medical routine	No intervention, standard care

Beurskens, 2007, Netherlands [25]	9 sessions of physiotherapy (once or twice a week for the first 3 weeks, and thereafter once a fortnight or less). 90 sessions of home exercises of 10 min (once a day)	Physiotherapy: soft tissue massage of the surgical scar; exercise for arm/shoulder, muscular strength, coordination, and improvement of general physical condition. Home exercises: exercises for the arm/shoulder	90 sessions of home exercises of 10 min (once a day)	Home exercises: exercises for the arm/shoulder

Wilkie, 2000, USA [27]	4 sessions of massage therapy of 30–45 min (twice a week)	Massage therapy: massage of head/back/gluteus muscles/four extremities, including effleurage, light petrissage, naive stroke, light compression, vibration, and tapotement	Medical routine	No intervention, standard care

Wilkinson, 2007, UK [28]	40 sessions of aromatherapy massage of 60 min (4 times a week)	Aromatherapy massage: massage with essential oils, massage strokes, timings, and overall style	Usual supportive care	Usual supportive care: psychological support services

Collinge, 2013, USA [29]	12 sessions of massage therapy of 20 min (3 times a week)	Massage therapy: manual techniques for comfort and relaxation of head/neck/shoulders/back/feet/hands, including touching and acupressure	12 sessions of reading therapy of 20 min (3 times a week)	Reading therapy: reading any literature such as poetry, fiction, nonfiction, and religious

Pyszora, 2017, Poland [30]	6 sessions of physiotherapy programme of 30 min (3 times a week)	Physiotherapy programme: techniques of myofascial release and proprioceptive neuromuscular facilitation	Medical routine	No intervention, standard care

Arienti, 2018, Italy [31]	4 sessions of osteopathic manipulative treatment of 45 min (once a week). 28 sessions of physiotherapy of 30 min (once a day)	Osteopathic manipulative treatment: dorsal/lumbar soft tissue/rib raising; back/abdominal myofascial release; cervical spine soft tissue/suboccipital decompression; sacroiliac myofascial release; strain-counterstain; and muscle energy technique. Physiotherapy: passive mobilization, active exercises, and walk	28 sessions of physiotherapy of 30 min (once a day)	Physiotherapy: passive mobilization, active exercises, and walk

Qian, 2018, China [32]	28 sessions of massage therapy of 10 min (twice a day)	Massage therapy: pressing and rubbing with oils on Baihui (DU20)/Shenmen (HT7) and other acupoints related to symptoms	Medical routine	No intervention, standard care

Chen, 2019, China [33]	28 sessions of acupressure of 20 min (twice a day)	Acupressure: pressing on Neiguan (PC6) and Zusanli (ST36)	Medical routine	No intervention, standard care

EG: experiment group; CG: control group.

**Table 3:** PEDro scale of methodological quality assessment for the studies.

First author	Eligibility criteria	Random allocation	Concealed allocation	Similar at baseline	Subjects blinded	Therapists blinded	Assessors blinded	<15% dropouts	Intention-to-treat analysis	Between-group comparisons	Point measures and variability data	Total
De Groef [14]	1	1	1	1	1	0	1	1	1	1	1	9
Listing [15]	1	1	0	1	0	0	1	0	1	1	1	6
Rangon [24]	1	1	1	1	0	0	1	1	0	1	1	7
Wyatt [13]	1	1	1	1	0	0	1	1	0	1	1	7
Tsay [26]	1	1	1	1	0	0	1	1	0	1	1	7
Beurskens [25]	1	1	1	1	0	0	1	1	0	1	1	7
Wilkie [27]	1	1	0	1	0	0	1	1	0	1	1	6
Collinge [29]	1	1	0	1	0	0	1	1	1	1	1	7
Wilkinson [28]	1	1	1	1	0	0	1	1	0	1	1	7
Pyszora [30]	1	1	1	1	0	0	1	1	0	1	1	7
Arienti [31]	1	1	0	1	0	0	1	1	0	1	1	6
Qian [32]	1	1	0	1	0	0	1	1	0	1	1	6
Chen [33]	1	1	0	1	0	0	1	1	0	1	1	6

PEDro: physiotherapy evidence database. Criteria (2–11) were used to calculate the total PEDro score. Each criterion was scored as either 1 or 0 according to whether the criteria was met or not, respectively.

## Data Availability

The data used to support the findings of this study are available from public databases, and more details also can be obtained from the corresponding author on request. He can be reached at fm-tn0510@shutcm.edu.cn.
